# Enhancing autonomous exploration for robotics via real time map optimization and improved frontier costs

**DOI:** 10.1038/s41598-025-97231-9

**Published:** 2025-04-10

**Authors:** Chunyang Liu, Dingfa Zhang, Weitao Liu, Xin Sui, Yan Huang, Xiqiang Ma, Xiaokang Yang, Xiao Wang

**Affiliations:** 1 Henan University of Science and Technology, Luoyang, 471000 Henan Province China; 2 Longmen Laboratory, Luoyang, 471000 China; 3 Key Laboratory of Mechanical Design and Transmission System of Henan Province, Luoyang, 471000 China; 4 Collaborative Innovation Center of Machinery Equipment Advanced Manufacturing of Henan Province, Luoyang, 471000 China

**Keywords:** Wheeled robots, Mapping, Autonomous exploration, Engineering, Electrical and electronic engineering

## Abstract

Autonomous exploration and mapping in unknown environments remain pivotal in robotics research. The efficiency of autonomous exploration is often constrained by irrational exploration strategies and incomplete map exploration. This paper proposes an efficient autonomous exploration method based on a frontier strategy, aiming to enhance the performance of ground mobile robots in exploration and mapping tasks. We employ a real-time grid map optimization technique using bilateral filtering and expansion to eliminate inefficient frontiers, improve mapping quality, and enhance the overall efficiency of autonomous exploration. Additionally, we construct a novel frontier cost function that incorporates factors such as path length, sensor measurement range, and information gain. Our approach uniquely combines an autonomous exploration decision model with the Minimum Ratio Travelling Salesman Problem (MRTSP) to maximize the explored area within the shortest possible path. Comparative analyses with classic methods, conducted in both simulated and real environments, demonstrate a 10–30% improvement in exploration efficiency through our approach.

## Introduction

With the increasing fusion of intelligence and robotics, autonomous mobile robots are rapidly gaining prominence across various industries. Autonomous exploration (AE) is a critical method for robots to perceive and map in 2D environments, providing foundational information for autonomous navigation. The extensive applications of autonomous mobile robots include disaster detection and rescue^1^, substation^2^, autonomous driving^3^, and logistics warehouse, marking AE technology as a cornerstone of intelligent autonomous systems.

In recent years, numerous scholars have delved into AE research.The state-of-art AE approaches include frontier-based AE, sampling-based AE, Next-Best-View (NBV), and machine learning-based AE. The NBV method is a widely utilized 3D exploration and mapping approach that iteratively identifies viewpoints with the richest perceptual information. Machine learning approaches perform well at the costs of substantial computational resources and extensive training times. For 2D mapping and exploration, sampling-based AE and frontier-based AE are more favorable choices regarding their high exploration efficiency. Sampling-based approaches, such as the Rapidly Expanding Random Tree (RRT), utilize randomized sampling to cluster sample points and obtain exploration targets. However, due to their instinctive randomness, sampling-based AEs might select inefficient exploration targets and compromise the overall strategy’s efficiency^4^ Frontier-based AEs establish exploration targets by detecting the junction between explored and unexplored regions in unknown environments. As target selection approaches more effectively than sampling-based AEs, the frontier-based AEs remove inefficient exploration paths caused by random sampling and optimize subsequent target selection by utilizing explored environmental information. Thus, this paper selects the frontier-based method for mobile robot navigation.

Further, edge detection and region extraction techniques are commonly used in frontier-based AEs to promote frontier detection, where the detection accuracy is mainly determined by the quality of map construction. Invalid or inaccurate frontiers could introduce a significant amount of noise into the established map. In real-time applications, the computational complexity of MRTSP increases exponentially with the number of frontier points, making it challenging to meet the processing speed requirements of real-time systems. Building on this, the current study adopts and updates a greedy strategy-based algorithm to locate optimal target points, aiming to escape from suboptimal local minima^5^ and fulfill efficient autonomous exploration by accounting for global information. In short, the contributions of this paper are summarized as follows:

1. Bilateral filtering, combined with dilation, is employed to optimize the grid map in real-time. The updated approach enhances the quality of the 2D map by reducing the likelihood of reappearance of established frontiers in explored areas. The proposed approach is validated to promote the decision speed and success rate of autonomous exploration accordingly.
2. Constructing a comprehensive frontier cost function that incorporates key factors such as path length, sensor measurement range, and information gain. The cost function transforms the AE task into a Minimum Ratio Travelling Salesman Problem (MRTSP) and helps solve the optimal exploration sequence.
3. Conducting multiple quantitative comparisons in simulations and experimental environments. The comparison confirms the practicality and engineering applicability of our method in various environments.

## Related work

In 1997, Yamauchi^6^ defined the frontier in map exploration as the interface between known and unknown regions. Since then, frontier-based AE methods have continuously been updated with the advances in frontier detection and exploration strategies.

Frontier detection is the primary task in frontier-based AEs, and its speed significantly impacts the overall efficiency. To address the slow efficiency of traditional frontier detection methods^7,8^, two approaches to accelerate frontier detection speed have been proposed^9^, namely Fast Frontier Detector (FFD) and Wavefront Frontier Detector (WFD). The FFD mainly relies on real-time sensor data to extract new frontier points, and the Sparse Rough Frontier Point Generation^10^ is applied to refine frontiers by integrating a global topology map. An alternative approach^11^ crops frontiers within active regions to reduce the computational burden of frontier detection. On the other hand, WFD utilizes the breadth-first search strategy to locate frontier points. The Expanded-Wavefront Frontier Detection (EWFD)^12^ uses the frontier points of the previous time step as the starting point for the current time step in searching the frontier cells. The Frontier-Tracing Frontier Detection^13^ promoted EWFD by optimizing the search time step based on historically scanned areas. However, the practical efficiency of the above methods is susceptible to the impact of inefficient frontier. The accumulation of inefficient frontiers during exploration leads to a cumulative effect that significantly increases the computational cost and compromises the accuracy of frontier exploration.

Exploration strategy also significantly affects AEs’ efficiency, where the essential tasks include cost function construction and solving technique selection. The cost function quantifies the exploration cost using multiple parameters, such as information gain^14^, localization quality, navigation costs, and mutual information^15,16^. For example, TAD^17^ leverages the trapezoidal shape, adjacency relationships, and distance characteristics of frontiers. The Rmap^18^ algorithm dynamically determines inner and outer frontier pairs based on sensor range adjustments, calculates their lengths, and selects goal points for efficient exploration. Ref.^19^ constructs a cost function based on map uncertainty and robot posture. Next, the solving technique is consequently selected according to the forms of cost functions. Reported studies usually factored distance-based cost functions into Traveling Salesman Problems (TSPs)^20^ or constrained TSPs^21^, and employed generic evolutionary algorithms to determine the optimal exploration targets. In addition, various information, like the local exploration path step size, can accelerate the scoping of the optimal frontier points^22^. Recently, conventional frontier-based AEs have been innovatively combined with deep reinforcement learning to determine the following target points^23^. Based on the above discussion, this study constructs the cost function using information gain and path cost, for which the MRTSP-based solving technique dynamically determines the optimal target points.

**Fig. 1:**
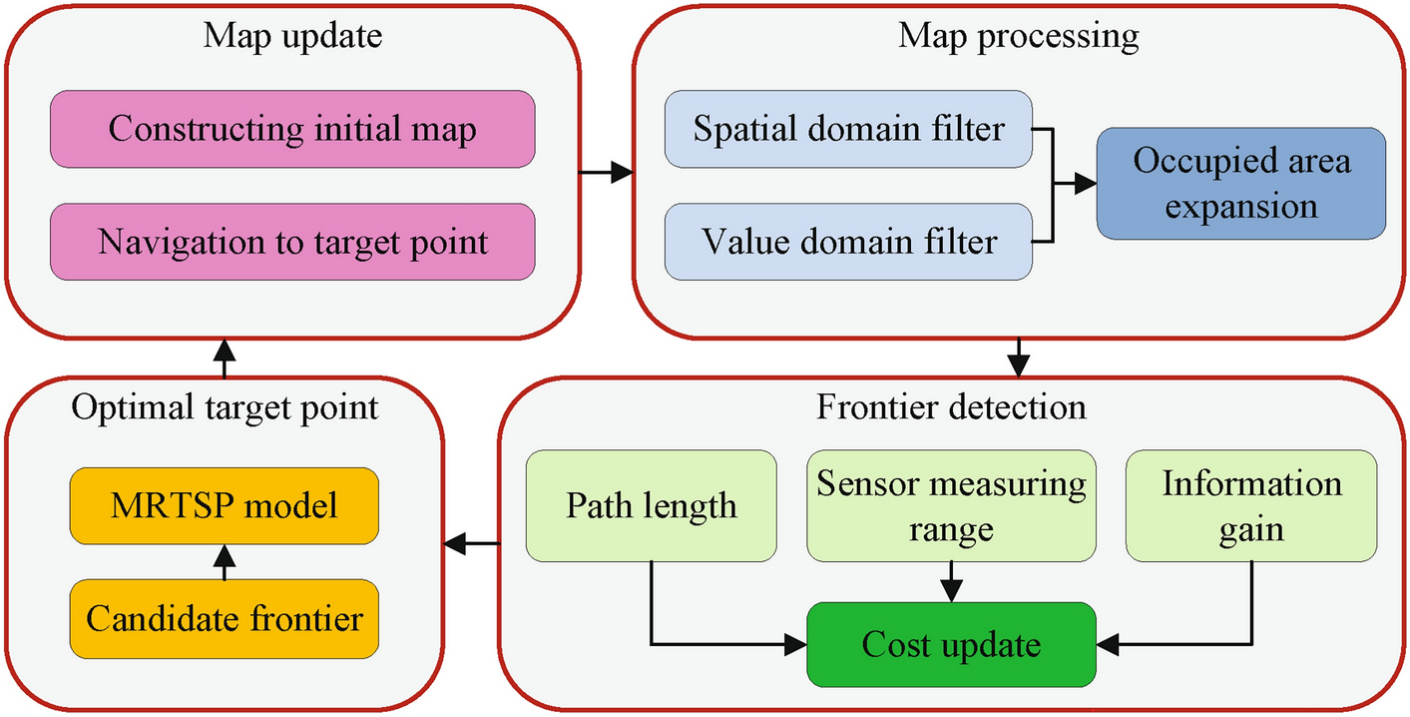
Autonomous exploration flowchart.

## Proposed algorithm

Our proposed autonomous exploration process is illustrated in Fig. 1. Initially, we construct and optimize a map using sensor data. Following this, frontier detection and cost evaluation are performed on the optimized map, where the frontier cost evaluation accounts for path length and sensor’s measurement range. We then establish an autonomous exploration model based on MRTSP to determine the optimal sequence of exploration targets. The robot navigates to these targets and updates the map. This process continuously explores unknown areas and constructs scene maps until no new frontiers emerges.

### Map optimization

**Fig. 2:**
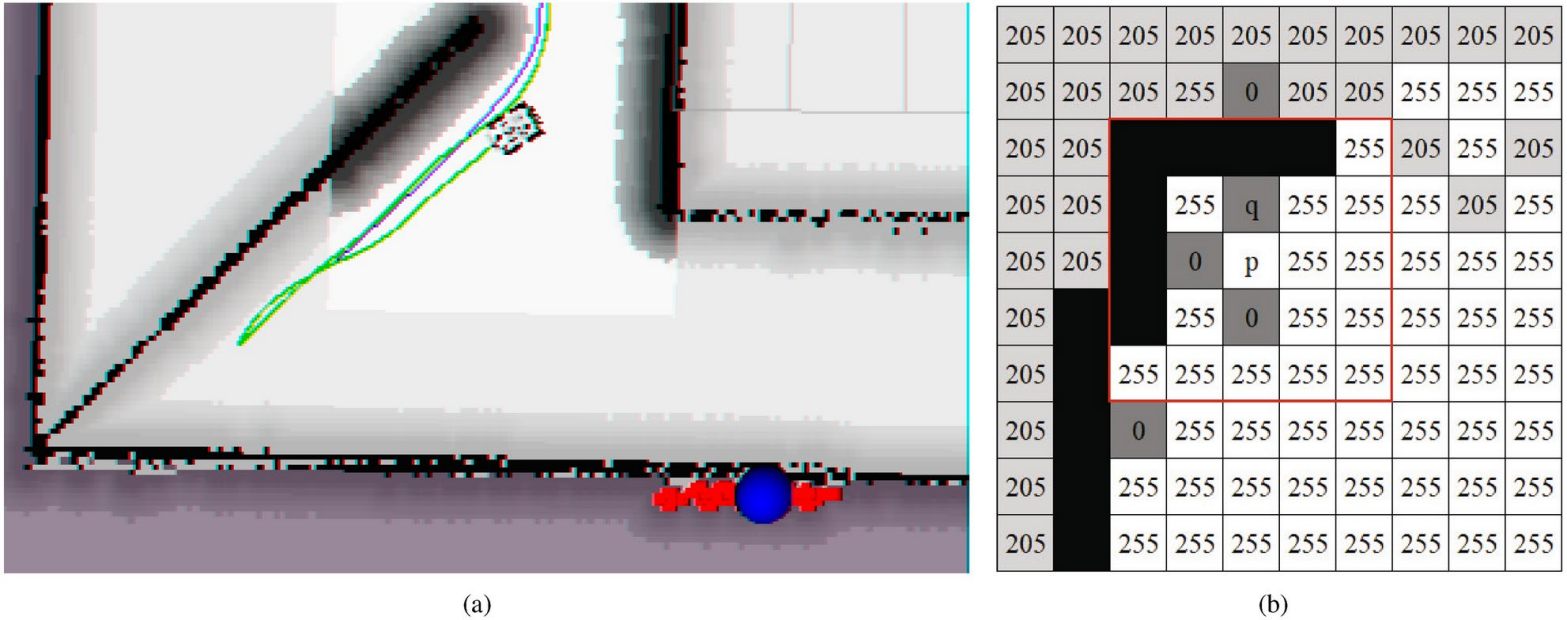
The figure on the left illustrates the inefficient frontier formed during the exploration process. The right shows the raster state after the initial map adjustment.

During the grid map update process, the precision and clarity of the map decrease with increasing distance from the lidar due to the sparsity of laser beams. As shown on the left side of Fig. 2, frontier structures can still be found within areas the robot has already explored. In such situations, the robot is prone to repeatedly exploring these areas and may even enter a pseudo-stagnant state. The solution time for the MRTSP is significantly affected by the number of frontiers. Therefore, we optimize the map to enhance mapping quality and reduce the number of frontiers.

In this paper, the grid map data is processed using bilateral filtering. To emphasize the edge structures on the map, occupied areas are strictly distinguished from other regions to signal the presence of frontier structures. Initial map data is adjusted accordingly to the reference image data. Furthermore, a spatial domain Gaussian function and a pixel domain Gaussian function are constructed within the grid coordinate system as the following:

1 $$\begin{aligned} & G_{\sigma s}(\Vert p-q\Vert )=e^{-\frac{(i-x)^2+(i-y)^2}{2\sigma ^2_s}} \end{aligned}$$

2 $$\begin{aligned} & G_{\sigma s}(\Vert p-q\Vert )=e^{-\frac{(i-x)^2+(i-y)^2}{2\sigma ^2_s}} \end{aligned}$$

In Fig. 2b, where q represents the input grid, x and y are the grid’s horizontal and vertical coordinates, respectively, p is the box’s central grid, i and j are p’s coordinates. $I_o(x, y)$ is the value of the input grid, while $I_c(i, j)$ represents the value of the central grid in the box. $\sigma _s$ and $\sigma _r$ are the spatial domain standard deviation and grey value domain standard deviation, respectively.

In Fig. 2b, the real obstacles are colored black. Unknown areas, fake obstacles (obstacles created by mistake), and known areas are assigned with values of 205, 0, and 255, respectively. Equation (2)’s value approaches 0 when the grid value changes significantly near a real obstacle, which reduces the grids’ impacts and preserves the impact of real obstacles. In Fig. 2b’s upper right corner, unknown areas created by sparse laser beams intersect with known areas, and Eq. (1) becomes larger as the grid value varies smoothly. Such a process, resulting in a similar result as applying the Gaussian blur operation to the region, can amend the wrongly defined unknown areas to known areas based on laser beam’s sparsity, effectively downsizing the number of frontiers.

Consequently, the parameter $\sigma _s$ in the spatial domain Gaussian function (Eq. 1) is determined based on the maximum distance between fake obstacles and real obstacles. The parameter $\sigma _r$ in the pixel domain Gaussian function (Eq. 2) is determined based on grid value changes at the transferring sections between known and unknown areas near real obstacles. These functions help retain the map’s edges and blur the invalid frontiers between known and unknown areas. With them, the bilateral filter is calculated as follows:

3 $$\begin{aligned} & W_p=\sum _{q\in s} G_{\sigma s}(\Vert p-q\Vert )G_{\sigma r}(|I(p)-I(q)|) \end{aligned}$$

4 $$\begin{aligned} & \bar{I}(p)=\frac{1}{W_p}(\sum _{q\in s} G_{\sigma s}(\Vert p-q\Vert )G_{\sigma r}(|I(p)-I(q)|)I(p)) \end{aligned}$$

When the filter’s window traverses over the map, every point covered by the window is connected to the window’s central point located at (i, j), with which Eq. (3) generates a corresponding sum Wp, and Eq. (4) updates the grid’s $\bar{I}(p)$, accordingly. The bilateral filtering process, while preserving the edge information near real obstacles, can create several unknown areas. Therefore, an expansion operation, following the bilateral filtering, is performed on explored regions to remove unknown areas. The expansion template’s size is determined by the range of the spatial domain Gaussian function (Eq. 1), and the operation improves the exploration efficiency by stressing the characteristics of obstacles and reducing invalid frontiers.

### Frontier detection

**Fig. 3:**
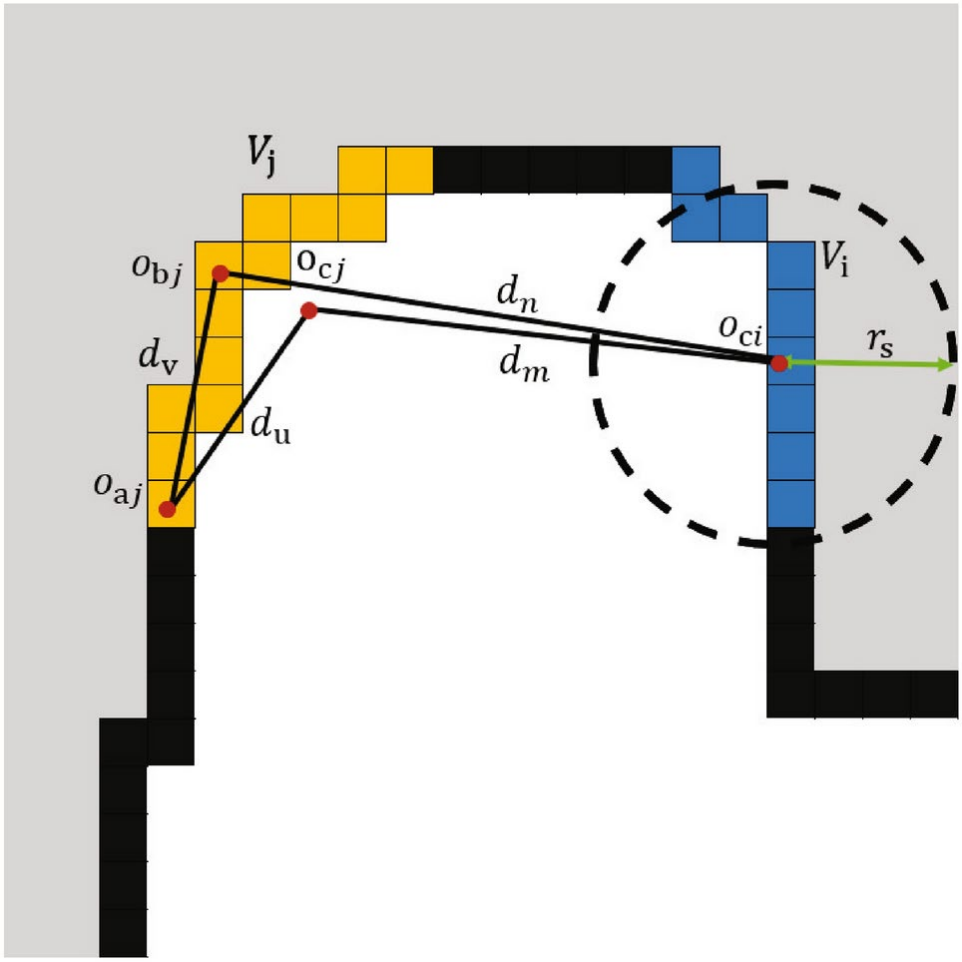
Schematic diagram of frontier cost construction: explored area (black), known areas (white), unknown areas (grey), and frontier $V_i$ (Blue) and $V_j$ (Yellow).

GMAPPING is used to construct 2D grid map during the robot exploration process, where WFD is employed to detect frontier. Inspired by FUEL^24^ [23], consecutive frontier cells form a frontier featuring multiple key parameters, such as the location of every included cell, starting cell, centroid, center point, path cost, and exploration gain of the frontier. For two frontiers $V_i$ and $V_j$ illustrated in Fig. 3, $O_{aj}$ is $V_j$’ starting point, $O_{bj}$ and $O_{cj}$ are the centroid and the center of frontiers Vi$V_i$ and $V_j$, respectively. dn and dm are distances from frontier $V_i$ to the centroid and center of frontier $V_j$, $d_v$ and $d_u$ are distances from frontier $V_i$’s centroid and center to the starting point $O_c$, respectively, $r_s$ is the effective detection range of the sensor. Thus, the path cost and exploration gain of a frontier are calculated as,

5 $$\begin{aligned} & d(V_i,V_j)=max(d_m+d_u,d_n+d_v)-r_s \end{aligned}$$

6 $$\begin{aligned} & P(V_i,V_j)=S \end{aligned}$$

where S is the size of frontier $V_j$. Equation (5) will produce a negative $d(V_i, V_j)$ if a frontier lies outside the sensor’s detection range, consequently prioritizing in-range frontiers for exploration. Therefore, the path cost $d(V_i, V_j)$ specifies the shortest path from $V_i$ to $V_j$ while ensuring frontier $V_j$ stay in the sensor’s detection range. Besides, frontier $V_j$’s size S is considered as the information gain $p(V_i, V_j)$ (Eq. 6).

The Yamauchi method employs a single distance metric, which is essentially a greedy algorithm that only considers the shortest path from the robot’s current position to the frontier point. In contrast, this paper proposes a multi-factor integrated cost function: it combines path length (Eq. 5), sensor range and information gain (Eq. 6). This multi-dimensional optimization framework allows for a more comprehensive evaluation of the exploration value of frontier points.

### Exploring sequence optimization

**Fig. 4:**
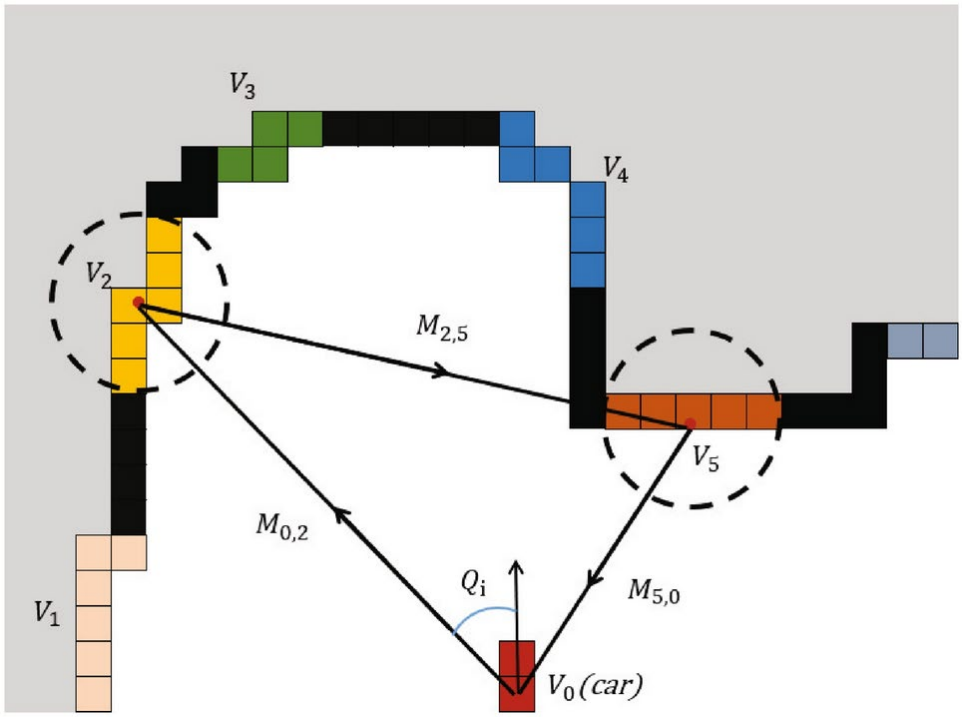
Demonstration of the MRTSP cost function construction process.

Once obtaining the path cost and information gain, MRTSP model will determines the optimal exploration sequence by minimizing the ratio of the length of the navigation path to total explored area. For a robot located at location $V_0$, a cost matrix M is constructed to quantify the ratio of path cost to exploration gain for all frontiers (Fig. 4). The matrix’s element $M_{i,j}$ is defined as,

7 $$\begin{aligned} M_{i,j} = {\left\{ \begin{array}{ll} \frac{W_d \times d(V_i, V_j)}{W_s \times P(V_i, V_j)} & \text {for } i \ne 0, j \ne 0 \quad \quad \text {(a)} \\ \frac{W_d \times d(V_i, V_j)}{W_s \times P(V_i, V_j)} + t_{lb}(V_i, V_j) & \text {for } i = 0, j \ne 0 \quad \quad \text {(b)} \\ 0 & \text {for } i \ne 0, j = 0 \quad \quad \text {(c)} \end{array}\right. } \end{aligned}$$

Three equations in Eq. (7) correspond to three cases:

1. Equation (7)a calculates the ratio of path cost to exploration gain for any two frontiers excluding the robot’s current location $V_0$, where Wd and Ws are weights for path cost and information gain, respectively.
2. Equation (7)b calculates the path cost and exploration gain based on the car’s current position $V_0$, the starting point for MRTSP. Besides the ratio as calculated in Eq. 7a, term tlb accounts for the cost from current position $V_0$ and any frontier $V_i$, such as,
  8 $$\begin{aligned} t_{lb}(V_i,V_j)=min\left\{ \frac{L(V_0,V_i)}{V_{max}}, \frac{min(|Q_i|,2\pi -|Q|)}{W_{max}} \right\} ,\ i=(1,...,n) \end{aligned}$$
  which represents the impact of the robot’s current speed and angle on the to-be-explore area $V_i$. In Eq. (8), $L(V_0, V_i)$ is the Euclidean distance between $V_0$ and $V_i$, $V_{max}$ is the robot’s maximum speed, $Q_j$ is the current robot to $V_j$ yaw angle, and $W_{max}$ is the maximum angular speed of the robot.
3. . Equation (7)c calculates the cost and gain from any Vi back to $V_0$, where set the element to zero to specify that the robot only needs to traverse all frontiers without returning to $V_0$.

Based on Eq. (7), Fig. 4 illustrates the matrix calculation for the robot’s current location $V_0$, and two candidate frontiers $V_2$ and $V_5$. After the MRTSP model determines the optimal frontier exploration sequence, the first frontier in the sequence is designated as the next target area for the robot to explore.

## Experiments and results

A four-wheel-drive robot is controlled based on the Robot Operating System (ROS). Gazebo is used to build models and simulation scenarios for testing. The tested model is further validated in real-world scenarios.

**Table 1:** Robot parameters in the experiment.

Parameters	Value
Robot size (cm)	41 $\times$ 34
Lidar mounting height (cm)	58.63
Lidar sampling range (rad)	$-3\sim 3$
Number of lidar samples	360
Lidar resolution	1
Lidar detection range (m)	$0.1\sim 30$
Linear velocity range (m/s)	$0.2\sim 0.5$
Angular velocity range (rad/s)	$-\,1\sim 1$
Linear acceleration (m/s^2^)	0.5
Angular acceleration (rad/s^2^)	0.8

**Table 2:** Set parameters for the experiment scenario.

Parameters	Sscenario		
	Corridor	Maze	Clutter
Size (m)	20$\times$34	20 $\times$ 20	15 $\times$ 15
Obstacle density (%)	1	9	7
Branching factor	7	9	13
Initial unknown ratio (%)	68	85	76

The computer for simulation test is equipped with an Intel i7 CPU, 16GB RAM, Ubuntu 18.04 OS with ROS Melodic, and a path planning provided by move_base^25^. The detailed simulation parameters are listed in Table 1. The main metrics^17^ used in the experiments are defined as follows:

**Exploration time:** The total time elapsed from the start of the task until the map coverage reaches $\ge$95% (including mapping, planning, and movement).

**Path length:** The actual length of the trajectory recorded by the robot’s odometer during the exploration time (not Euclidean distance).

**Exploration success rate:** The percentage of exploration attempts where the map coverage reaches 95% by the end of the exploration out of the total number of exploration attempts.

**Fig. 5:**
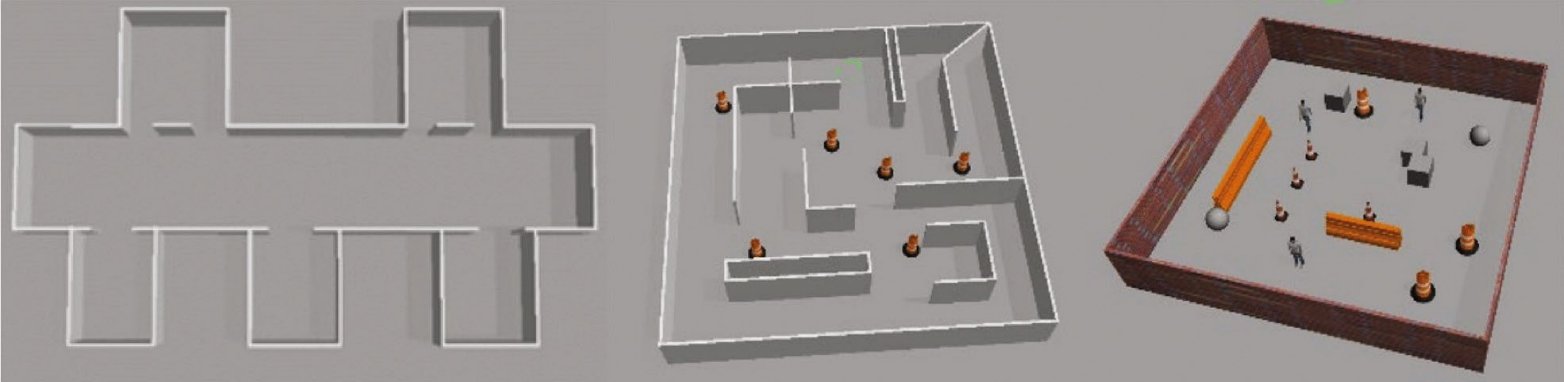
Simulated experimental scenario: (Left) corridor scenario (Center) maze scenario (Right) clutter scenario.

To validate our approach, we designed three scenarios as depicted in Fig. 5: corridor, maze, and cluttered environments. The corridor scenario represents a typical setup featuring a main corridor with multiple rooms branching off it. The maze scenario is designed with intricate paths and multiple intersections, making it ideal for testing exploration strategies. In contrast, the cluttered environment is filled with various obstacles, simulating a challenging condition. The map parameters (Table 2) are defined as follows:

**Obstacle density**^6^: The ratio of the area occupied by obstacles to the total area of the environment in a given map.

**Branching factor**^26^: The number of critical decision points in the environment, which refers to the total number of junctions where the robot needs to make path selection decisions.

**Initial unknown ratio**^27^: The ratio of unknown area relative to the total environment area at the start of the exploration task.

### Map optimization experiment

**Fig. 6:**
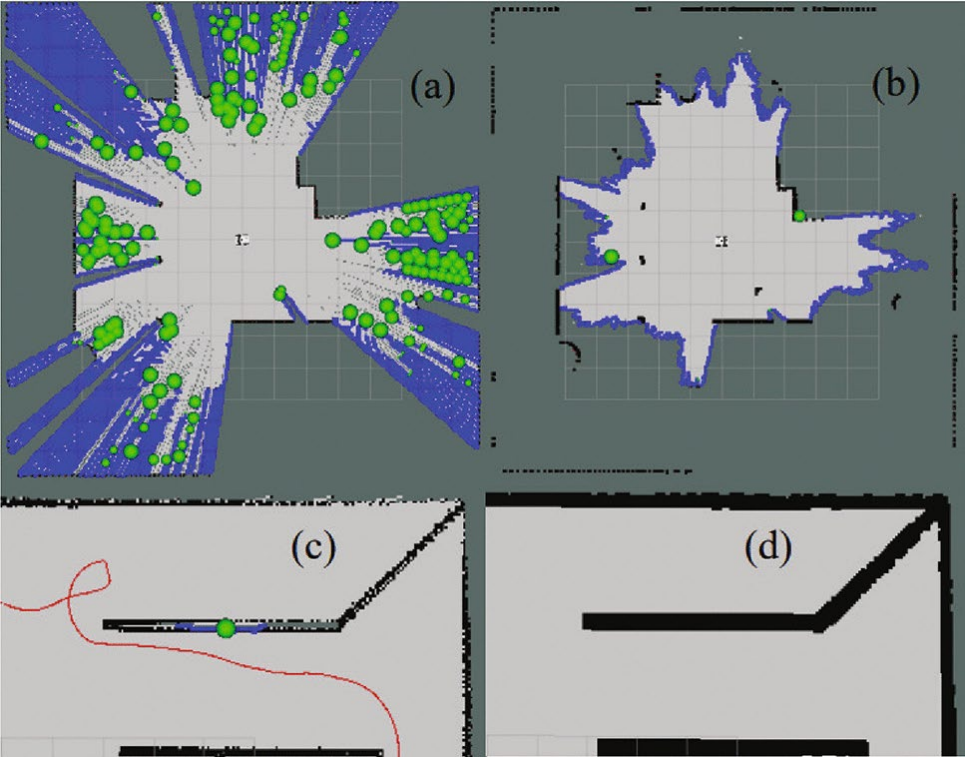
Comparison of the effect before and after map optimization.

Figure 6 demonstrates the effects of map optimization. Towards the far end of the laser radar’s detection range, laser beams become increasingly sparse, resulting in an excessive number of frontiers around the robot. The proposed real-time map optimization successfully reduces the number of frontiers during map construction (Fig. 6a and b). Simultaneously, this enhancement strengthens the map edges and prevents the generation of ineffective frontiers (Fig. 6c and d), thereby significantly improving the success rate of exploration.

**Fig. 7:**
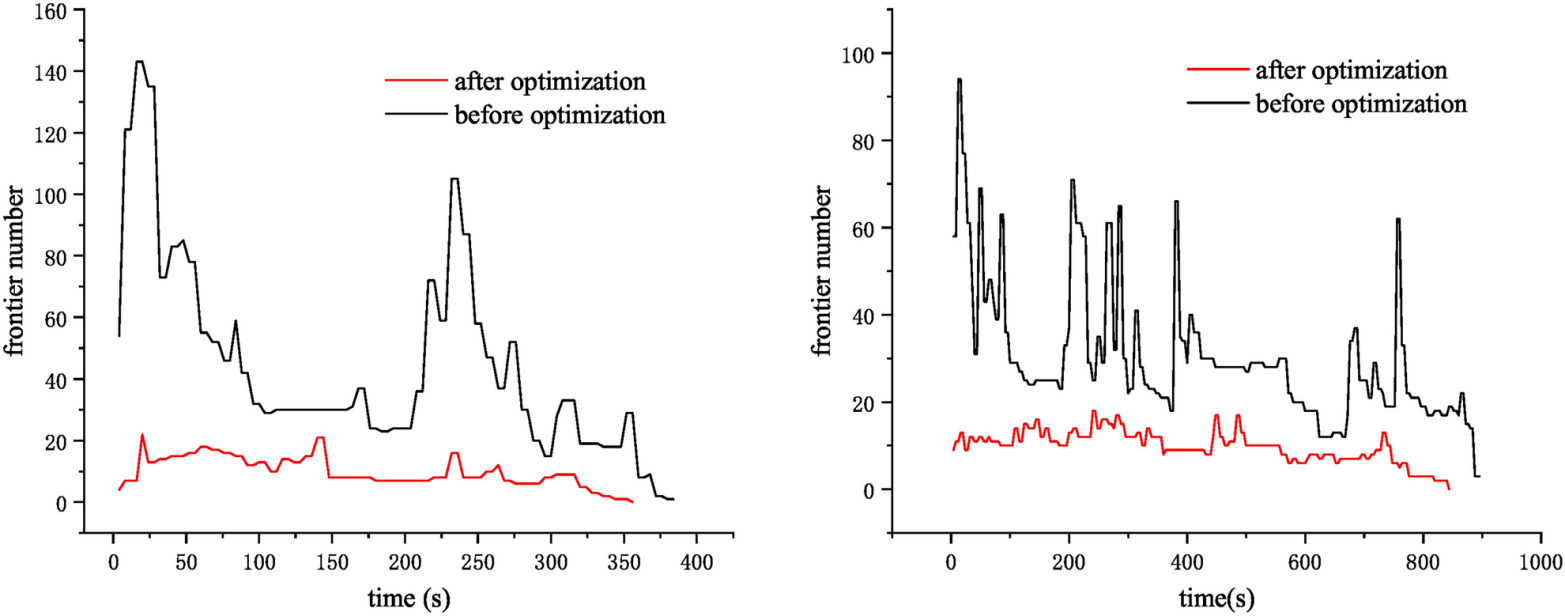
Comparison of the number of frontiers in two scenarios: corridor scenario (left) and maze scenario (right).

As a validation, we select two map scenarios (Fig. 5), namely ‘cluttered’ and ‘maze’, to test the proposed map optimization method. The frontier number downsizes remarkably after applying the proposed map optimization, (Fig. 7), which indirectly confirms that the method can enhance the efficiency of exploration.

### Exploration strategy comparison

Based on the benchmark method^28^, we made improvements to the exploration strategy, and therefore, We select two map scenarios from the previous map scenarios (Fig. 5), namely ‘maze’ and ‘corridor’, to test exploration strategy, in which our method and a benchmark method are tested ten rounds for the two map scenarios to eliminate randomness.

**Fig. 8:**
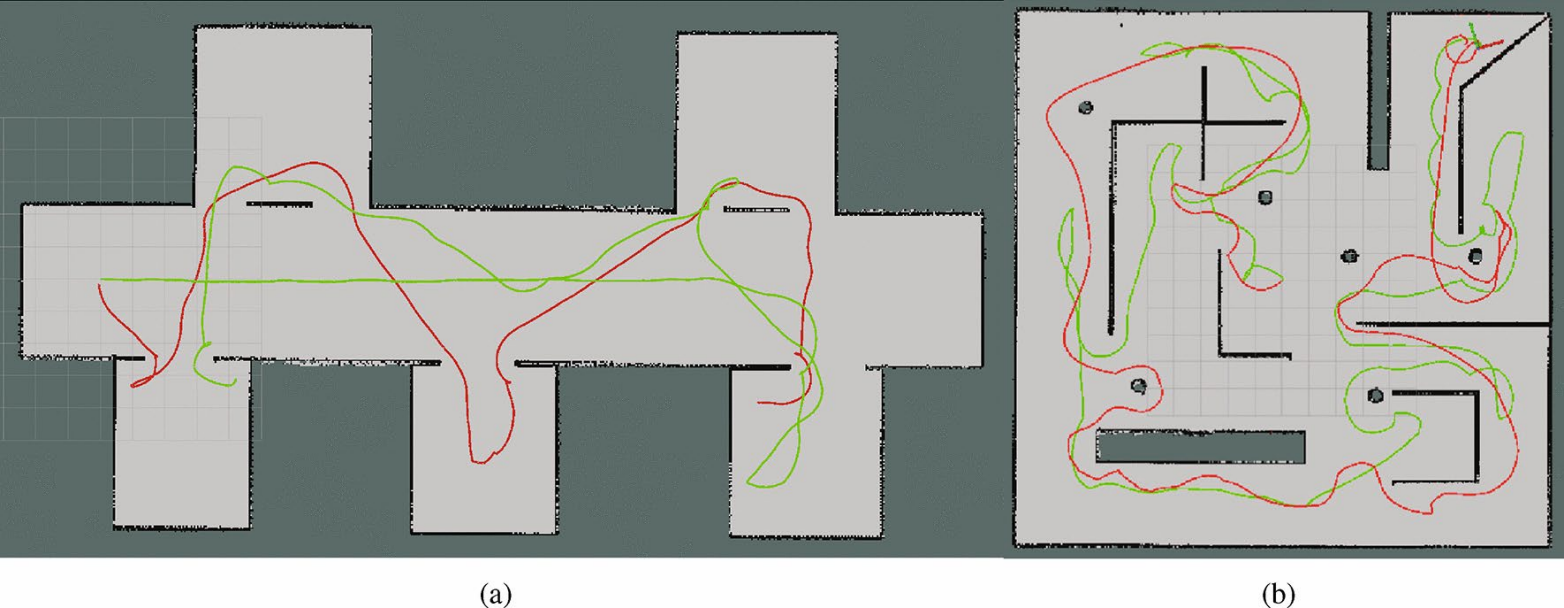
Maze and corridor scenarios and exploring experimental paths.

**Fig. 9:**
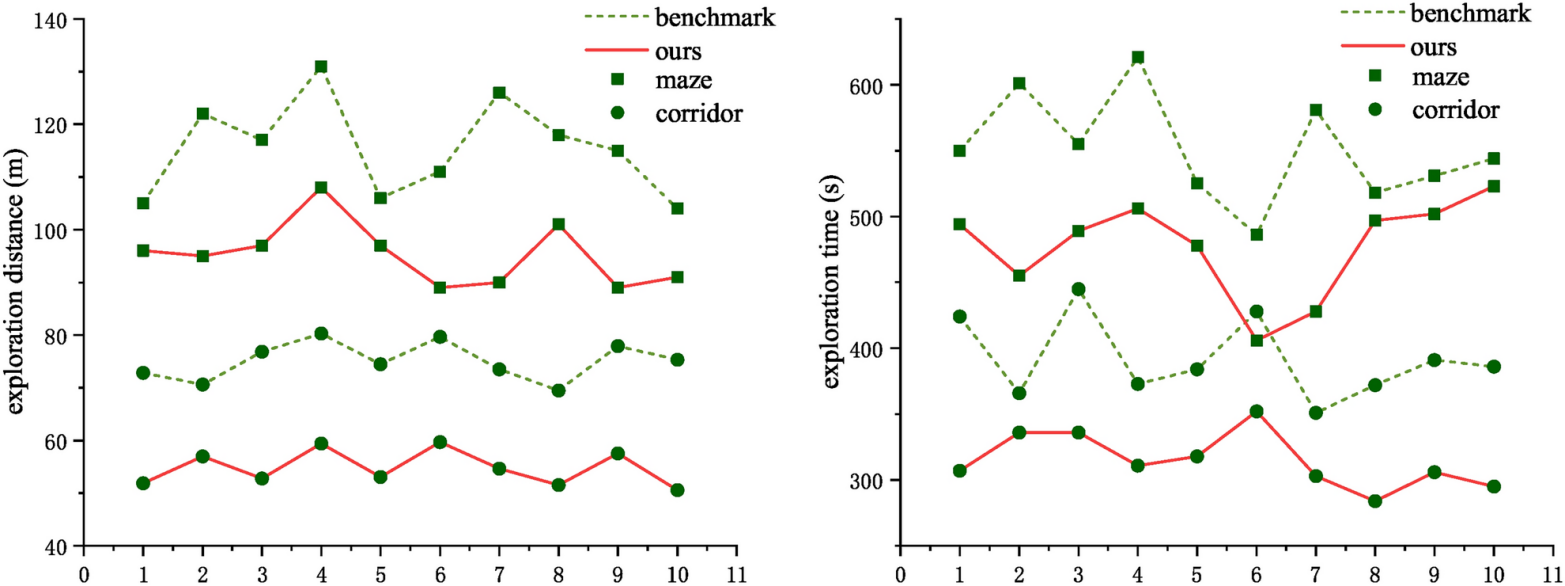
Exploring strategies experimental results (10 rounds).

In the corridor scenario (Fig. 8a), the benchmark method considers a frontier ahead as the optimal one and guides the robot to the corridor’s end before turning around for rooms in other directions, resulting in avoidable returns in the exploration. In contrast,our method introduces the MRTSP framework to achieve global path sequence optimization. This global optimization strategy effectively mitigates local optima issues, and its experimental outcome is reflected in the robot’s behavior of first exploring rooms on both sides until reaching the corridor’s end. As shown in Fig. 8b, compared to the benchmark, our method significantly reduces path complexity in the maze scenario, demonstrating enhanced exploration efficiency.

Experimental results (Fig. 9) show that our method reduces the path length by 27% and the exploration time by 19.69% on average in the corridor scenario and reduces the path length by 12.99% and the exploration time by 9.31% on average in the maze scenario.

### Comprehensive performance comparison

**Fig. 10:**
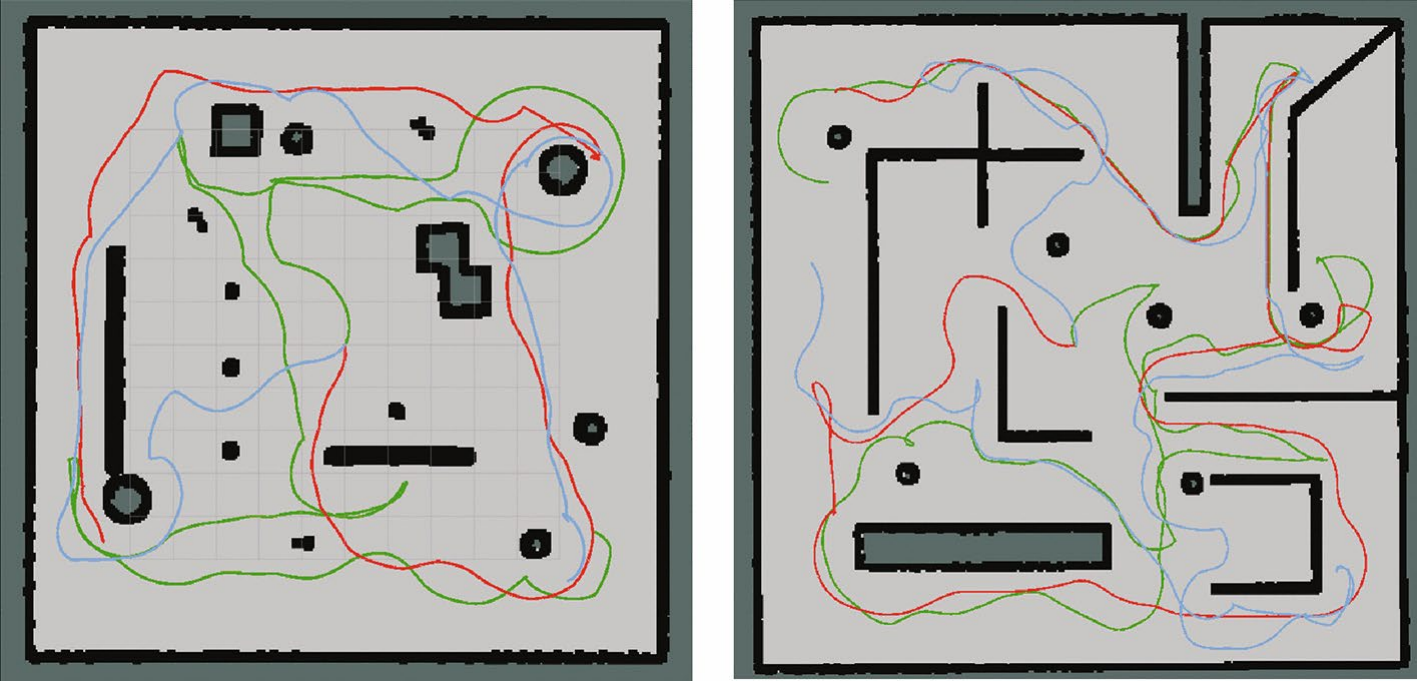
Experimental paths through Maze and cluttered scenes. Red path is our experimental path. Green path is the Nearest-frontier experimental path. Blue path is MSOD experimental path.

Next, we compare our method with RRT^29^, Nearest-frontier^6^, and an improved Nearest-frontier method based on Map Segmentation and Object Detection (MSOD)^30^ in three scenarios (Fig. 5), which include the corridor, a maze, and a cluttered setting. All methods have been tested ten times in the three scenarios (Fig. 5), and their exploration time and path are presented in Fig. 10 and Tab. 3 for comparison. When the experimental paths of the four methods are displayed on a single map, the image becomes cluttered. Therefore, we only show the experimental paths of the three frontier-based methods.

**Table 3:** Experimental results in three scenarios.

Method	Exploration time (s)				Exploration distance (m)				Success rate
	Avg	Std	Max	Min	Avg	Std	Max	Min	
Messye scenario: 20 m$\times$20 m									
RRT-exploration	315.2	19.76765034	343	286	53.536	6.288611806	57.29	50.29	60%
Nearest-frontier	281.5	15.770028	334	256	53.846	5.109608335	57.1	46.43	90%
MSOD	261.5	17.3334935	304	248	51.089	8.480414	69.77	40.43	80%
Ours	234.6	12.41128519	253	217	45.609	3.84379744	48.12	42.56	100%
Maze scenario: 20 m$\times$20 m									
RRT-exploration	502.2	17.38275007	526	474	111.676	8.578732074	125.87	99.96	70%
Nearest-frontier	437.1	18.220906	464	401	104.112	4.115708444	109.34	96.46	100%
MSOD	421.4	20.32241433	454	376	101.279	7.842671	115.92	91.43	100%
Ours	388.6	22.47754435	419	353	91.578	3.613708772	93.37	88.59	100%
Corridor scenario: 30 m $\times$ 22 m									
RRT-exploration	389.2	12.03162499	415	371	64.141	6.547326859	75	53.85	70%
Nearest-frontier	338.2	13.0207824	364	309	60.251	2.431955797	62.31	53.62	90%
MSOD	311.5	15.40941782	343	286	57.239	7.708733	77.27	49.22	100%
Ours	294.4	7.696752562	304	277	51.287	2.568945504	55.81	48.47	100%

**Fig. 11:**
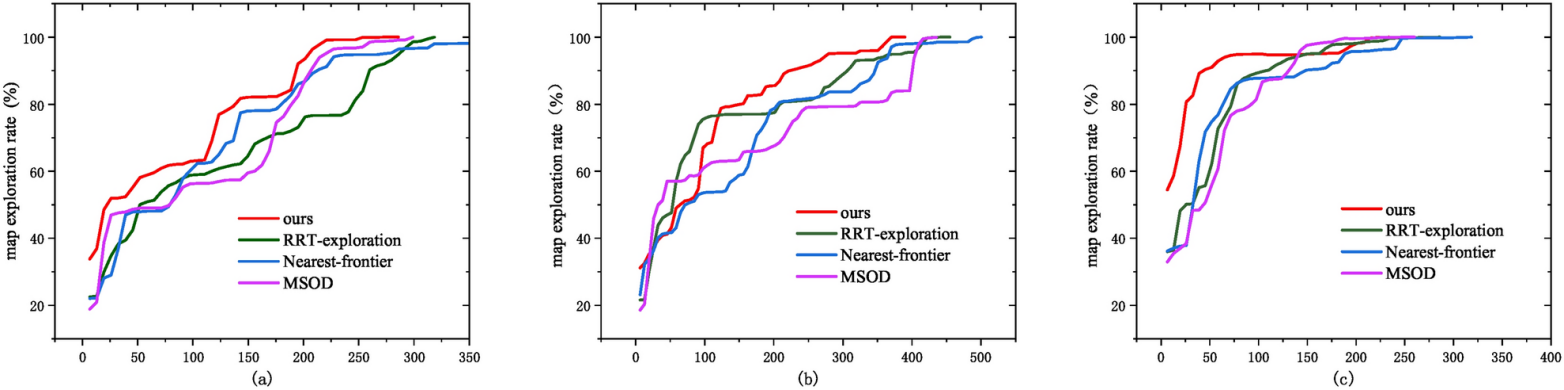
Comparison of the exploration process in three scenarios: (**a**) corridor scenario, (**b**) maze scenario, (**c**) clutter scenario.

Our method demonstrates superior performance in three scenarios compared to the other methods. As shown in Fig. 10, our exploration paths demonstrate fewer turns and repeated paths compared to those of MSOD and Nearest-frontier. The experimental results (Table 3) indicate that our method reduces the exploration time and path length by 31.93% and 21.46% compared to RRT-exploration. The exploration time and path length are also 5.78% and 16.40% shorter than the nearest-frontier. Also, our method outperforms MSOD by 8.57% and 11.40% in path length and exploration time, respectively. Figure 11 shows the comparison of the exploration efficiency of different methods in different scenarios, and is able to observe that the exploration efficiency of our method is higher than other methods. The above improvements confirm our method as a more efficient approach for autonomous exploration.

For a given environmental map, there theoretically exists an optimal exploration path length. When the exploration path lengths obtained from multiple experiments are closer to this optimal value, it indicates that the method can generate more stable and consistent exploration paths. This stability is specifically reflected in the fact that the proximity of the exploration path length to the theoretical optimal value directly correlates with the repeatability of the exploration path. In other words, when the exploration path length approaches the theoretical optimal value, the differences between the actual exploration paths generated in different experiments are significantly reduced. In multiple experiments across three scenarios, our method consistently exhibited a small standard deviation (Table 3), which not only demonstrates the robustness of our approach but also indicates that the experimental results are closer to the theoretical optimal path. As for the exploration’s success rates, Table 3 indicates that our method retrieves superior exploration efficiency in nearly all scenarios.

When facing larger exploration spaces, the computational complexity of MRTSP increases significantly with the growth in data scale. However, after map optimization, the number of frontier points is greatly reduced, making the advantages of the global optimization strategy more prominent.

**Fig. 12:**
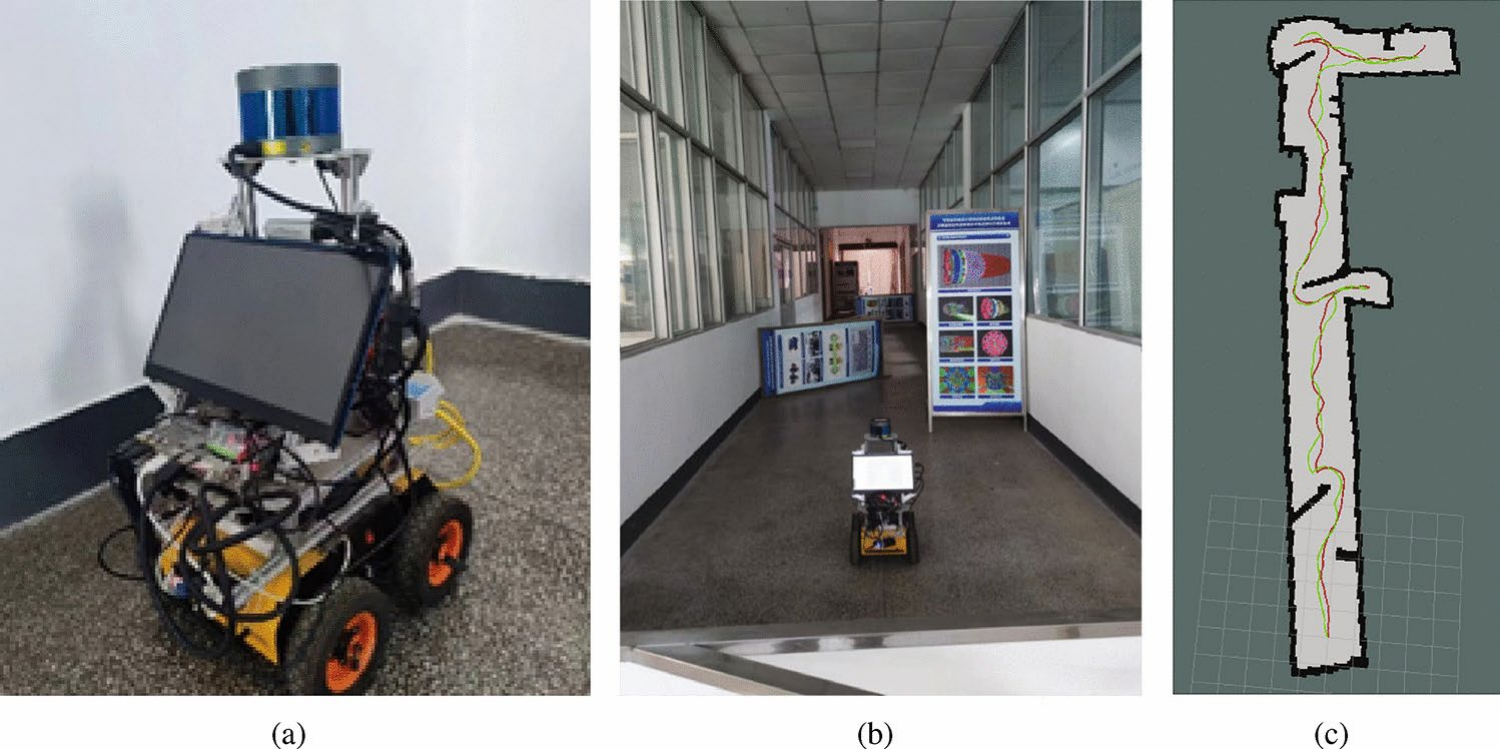
Real-world validation results: test robot (**a**), test scenario (**b**), and the exploration paths (**c**).

**Fig. 13:**
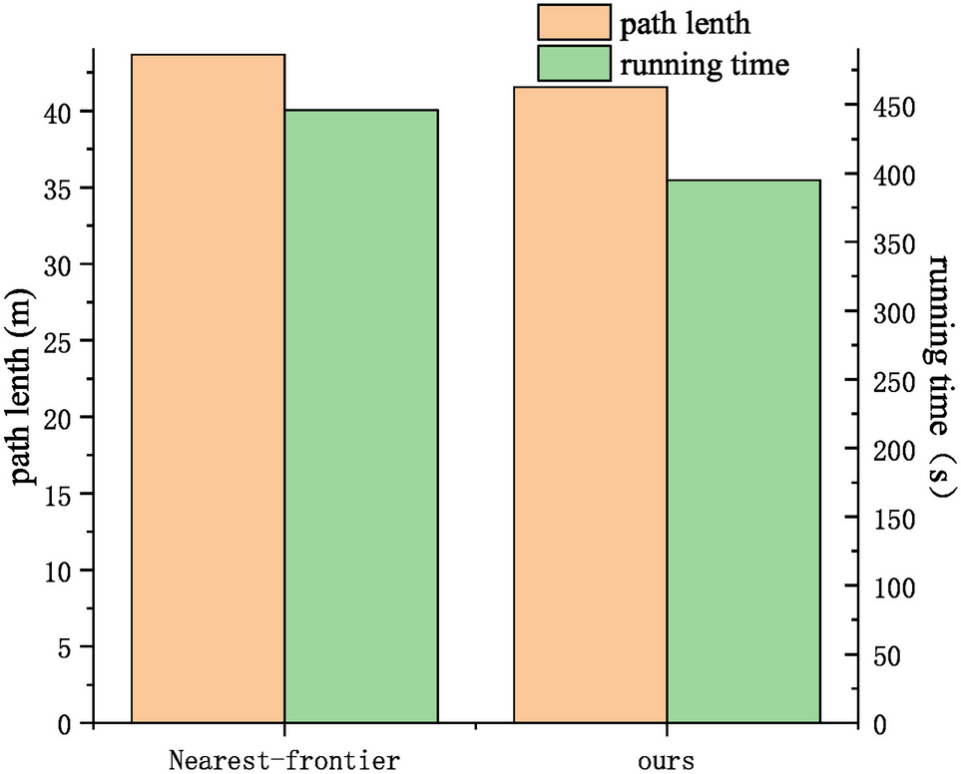
Real scenario experiment results.

### Real-world validation

In the simulation experiments, we validated the effectiveness of the method. However, due to the influence of environmental factors in real-world settings, we only verified the practicality and feasibility of our approach. In this validation, a four-wheel robot is employed as the platform to test multiple algorithms in real-world scenarios. The four-wheel robot (Fig. 12a) is equipped with a 16-line lidar, a Nvidia Jetson TX2 motherboard with preinstalled Ubuntu 18.04 OS and ROS Melodic. The real-world scenario is a L-shaped corridor (Fig. 12b) with multiple randomly placed obstacles (posters). For the two tested algorithms (the Nearest-frontier and ours), each one is tested for ten rounds to eliminate randomness. Their constructed map and exploration paths are depicted in Fig. 12c, for which the average exploration time and path length are compared in Fig. 13.

Compared to the traditional Nearest-frontier method, our method produces more successful navigation and yields shorter paths in the real-world environment. The method ensures successful exploration, while reducing the exploration time by 23.82% and the path length by 16.13%. Although the experimental setting is corridors, which are relatively regular environments, there are still some irregular corners. This causes Nearest-frontier to encounter false frontiers and expend additional time during exploration. Therefore, in the experimental results, despite the larger time comparison base, the time advantage remains greater than the path length advantage.

## Conclusion

This paper proposes an efficient approach for wheeled robots’ autonomous exploration and mapping. To reduce false frontiers caused by sparse laser beams, we combine bilateral filtering and dilation to enhance edge information during the map construction process. The proposed approach improves exploration efficiency by eliminating false frontiers and reducing the overall number of frontiers. thereby enhances the effectiveness in determining the optimal frontier exploration sequence. An improved frontier cost function is constructed accounting for multiple influencing factors, which helps locate an optimal exploring sequence based on MRTSP.

Experiment 1 validated the impact of map optimization on exploration efficiency. Experiment 2 confirmed that the improved cost function achieves better exploration objectives. Experiment 3 demonstrate that our method outperforms classical exploration methods, improving exploration efficiency by 10% to 30%. The real-world test further validates the effectiveness of our approach in practical applications. In future research, based on the MRTSP framework, autonomous exploration algorithms suitable for aerial and underwater robots could be developed by integrating the characteristics of three-dimensional environments and platform dynamic constraints.

## Data Availability

The availability of the raw data and code used in this study is limited. If you need data related to the results of the experiment, please contact Dingfa Zhang (220320010016@stu.haust.edu.cn).
